# Uncovering the neuroprotective effect of vitamin B12 in pneumococcal meningitis: insights into its pleiotropic mode of action at the transcriptional level

**DOI:** 10.3389/fimmu.2023.1250055

**Published:** 2023-10-03

**Authors:** Larissa Marcely Gomes Cassiano, Marina da Silva Oliveira, Karina Barbosa de Queiroz, Alice Muglia Thomaz da Silva Amancio, Anna Christina de Matos Salim, Gabriel da Rocha Fernandes, Cláudia Martins Carneiro, Roney Santos Coimbra

**Affiliations:** ^1^ Neurogenômica, Imunopatologia, Instituto René Rachou (IRR), Fiocruz, Belo Horizonte, MG, Brazil; ^2^ Departamento de Bioquímica e Imunologia, Universidade Federal de Minas Gerais, Belo Horizonte, MG, Brazil; ^3^ Plataforma Tecnológica de Sequenciamento NGS (Next Generation Sequencing), Instituto René Rachou (IRR), Fiocruz, Belo Horizonte, MG, Brazil; ^4^ Plataforma Tecnológica de Bioinformática, Instituto René Rachou (IRR), Fiocruz, Belo Horizonte, MG, Brazil; ^5^ Laboratório de Imunopatologia, Núcleo de Pesquisas em Ciências Biológicas, Universidade Federal de Ouro Preto, Ouro Preto, MG, Brazil

**Keywords:** pneumococcal meningitis, vitamin B12, epidrugs, neuroinflammation, histone methylation, neuroprotection

## Abstract

**Background:**

The interplay between bacterial virulence factors and the host innate immune response in pneumococcal meningitis (PM) can result in uncontrolled neuroinflammation, which is known to induce apoptotic death of progenitor cells and post-mitotic neurons in the hippocampal dentate gyrus, resulting in cognitive impairment. Vitamin B12 attenuates hippocampal damage and reduces the expression of some key inflammatory genes in PM, by acting as an epidrug that promotes DNA methylation, with increased production of S-adenosyl-methionine, the universal donor of methyl.

**Material and methods:**

Eleven-day-old rats were infected with *S. pneumoniae* via intracisternal injection and then administered either vitamin B12 or a placebo. After 24 hours of infection, the animals were euthanized, and apoptosis in the hippocampal dentate gyrus, microglia activation, and the inflammatory infiltrate were quantified in one brain hemisphere. The other hemisphere was used for RNA-Seq and RT-qPCR analysis.

**Results:**

In this study, adjuvant therapy with B12 was found to modulate the hippocampal transcriptional signature induced by PM in infant rats, mitigating the effects of the disease in canonical pathways related to the recognition of pathogens by immune cells, signaling via NF-kB, production of pro-inflammatory cytokines, migration of peripheral leukocytes into the central nervous system, and production of reactive species. Phenotypic analysis revealed that B12 effectively inhibited microglia activation in the hippocampus and reduced the inflammatory infiltrate in the central nervous system of the infected animals. These pleiotropic transcriptional effects of B12 that lead to neuroprotection are partly regulated by alterations in histone methylation markings. No adverse effects of B12 were predicted or observed, reinforcing the well-established safety profile of this epidrug.

**Conclusion:**

B12 effectively mitigates the impact of PM on pivotal neuroinflammatory pathways. This leads to reduced microglia activation and inflammatory infiltrate within the central nervous system, resulting in the attenuation of hippocampal damage. The anti-inflammatory and neuroprotective effects of B12 involve the modulation of histone markings in hippocampal neural cells.

## 1 Introduction

Acute bacterial meningitis (BM) is a severe and potentially life-threatening infection of the central nervous system (CNS). Currently, there is no specific treatment to prevent or reverse hippocampal damage caused by BM. *Streptococcus pneumoniae* is the leading etiological agent of community-acquired BM in children under 5 years old, and the elderly worldwide. Despite the availability of effective antibiotics, pneumococcal meningitis (PM) still carries significant morbidity and mortality rates (1). One of the most critical complications of BM is hippocampal damage, which can lead to severe cognitive deficits in survivors. The outcome of PM depends on complex interactions between the bacterial pathogenicity factors and the host response, including the activation of various immune signaling pathways (2).

Recent studies have shown that epigenetic modifications play a critical role in regulating the host response to PM. Epigenetic modifications refer to changes in gene expression mediated by non-coding RNA, DNA methylation, and histone modifications that do not involve alterations in the DNA sequence. Specifically, it has been demonstrated that global changes in the miRNome (3) and a decrease in global DNA methylation (4) occur in the hippocampus during PM. These alterations can impact genome stability and the expression of genes associated with neuroinflammation, oxidative stress, and the equilibrium between neurogenesis and neuronal death. As a result, epigenetic mechanisms can influence the infection’s outcome and contribute to the development of cognitive deficits in survivors. No prior research has explored the role of histone markings in modulating the host response to BM. Understanding the epigenetic regulation of the host response to PM has significant implications for the development of new therapies. Targeting specific epigenetic modifications may provide a novel approach to modulating the host response to infection, improving patient outcomes, and reducing the risk of long-term sequelae.

We have previously demonstrated that vitamin B12, a cofactor of the enzyme methionine synthase in the sulfur amino acid pathway, acts as an epidrug by increasing the production of S-adenosyl-methionine, the universal donor of methyl, in the hippocampus of infant rats with PM. By this mechanism, adjuvant therapy with B12 down-regulated the inflammatory genes Interleukin-1 beta (*Il1b*), C-C Motif Chemokine Receptor 2 (*Ccr2*) and C-C Motif Chemokine Ligand 3 (*Ccl3*) and largely attenuated the apoptotic death of progenitor cells and postmitotic neurons in the hippocampus dentate gyrus (4). Here, the safe profile of vitamin B12 and its positive effect on the hippocampal transcriptome of infant rats with PM are described, and evidence is provided that B12 also modulates methyl-dependent histone modifications.

## 2 Materials and methods

### 2.1 Animal model and experimental design

This study was approved by the Ethics Committee on the Care and Use of Laboratory Animals (CEUA-FIOCRUZ, protocol LW-23/17). An established experimental model of PM in infant rats was employed, following previously described procedures (4). Briefly, 11-day-old Wistar rats (20 ± 2 g) were infected by intracisternal injection of 10 μL saline (0.85% NaCl) containing ~2 × 10^6^ cfu/mL of *S. pneumoniae* (serotype 3, strain 38/12 MEN from the certified bacterial collection of the Ezequiel Dias Foundation). Sham-infected animals were injected with 10 μL of sterile saline. As at 11 days of age the rats have not yet entered puberty, their sex does not influence the parameters evaluated in this study, and therefore, half of the animals used were males and half were females. Animals were separated into four groups according to the treatment received: 10 μL of intramuscular vitamin B12 (Merck, Kenilworth, NJ; 0.065 mg/kg) (N = 10 infected + vitamin B12; N = 7 sham-infected + vitamin B12); or 10 μL of intramuscular saline (N = 6 infected + saline; N = 6 sham-infected + saline). Vitamin B12 and saline were administered at three and 18 hours post-infection. Eighteen hours after infection, all rats were clinically assessed using a stablished activity score (5) and infection was documented by quantitative culture of cerebrospinal fluid (CSF). Subsequently, all animals received 100 mg of ceftriaxone per kg of body weight administered subcutaneously (EMS Sigma Pharma Ltda., São Paulo, Brazil). Twenty-four hours post-infection, rats were euthanized by intraperitoneal overdose of ketamine (300 mg/kg) + xylazine (30 mg/kg) (Syntec, São Paulo, Brazil) and perfused via the left cardiac ventricle with 7.5 mL of RNAse-free ice-cold phosphate buffered saline (PBS). The brains were extracted from the skulls, and the two hemispheres were separated. The right hemispheres were fixed in 4% paraformaldehyde (PFA) (Sigma-Aldrich, St. Louis, MI) and further processed for histopathological assessment and immunohistochemistry analysis. The hippocampi from the left hemisphere were dissected and stored in RNAlater (Thermo Fisher, Waltham, MA) (for 24 hours at 4°C, followed by -80°C until use).

### 2.2 Brain histopathological analysis

To document the neuroprotective effect of vitamin B12 adjuvant therapy, the previously fixed right hemispheres of the brains were embedded in paraffin and cut into 5 µm-thick coronal sections using a microtome (Leica CM1850, Wetzlar, Germany). These sections were then stained with Cresyl violet and mounted onto microscope slides for observation under optical microscopy with a 40X objective and 10X eyepiece. The dentate gyrus’s lower blade was evaluated, and any neurons displaying morphological features indicative of apoptosis, such as condensed or fragmented nuclei and apoptotic bodies, were quantified in the largest visual field of four sections per rat. An average score per animal was determined from all the sections evaluated using a scoring system where 0–5 cells were given a score of 0, 6–20 cells were scored as 1, and >20 cells received a score of 2 (3, 4).

### 2.3 Transcriptome analysis

**Figure 1:**
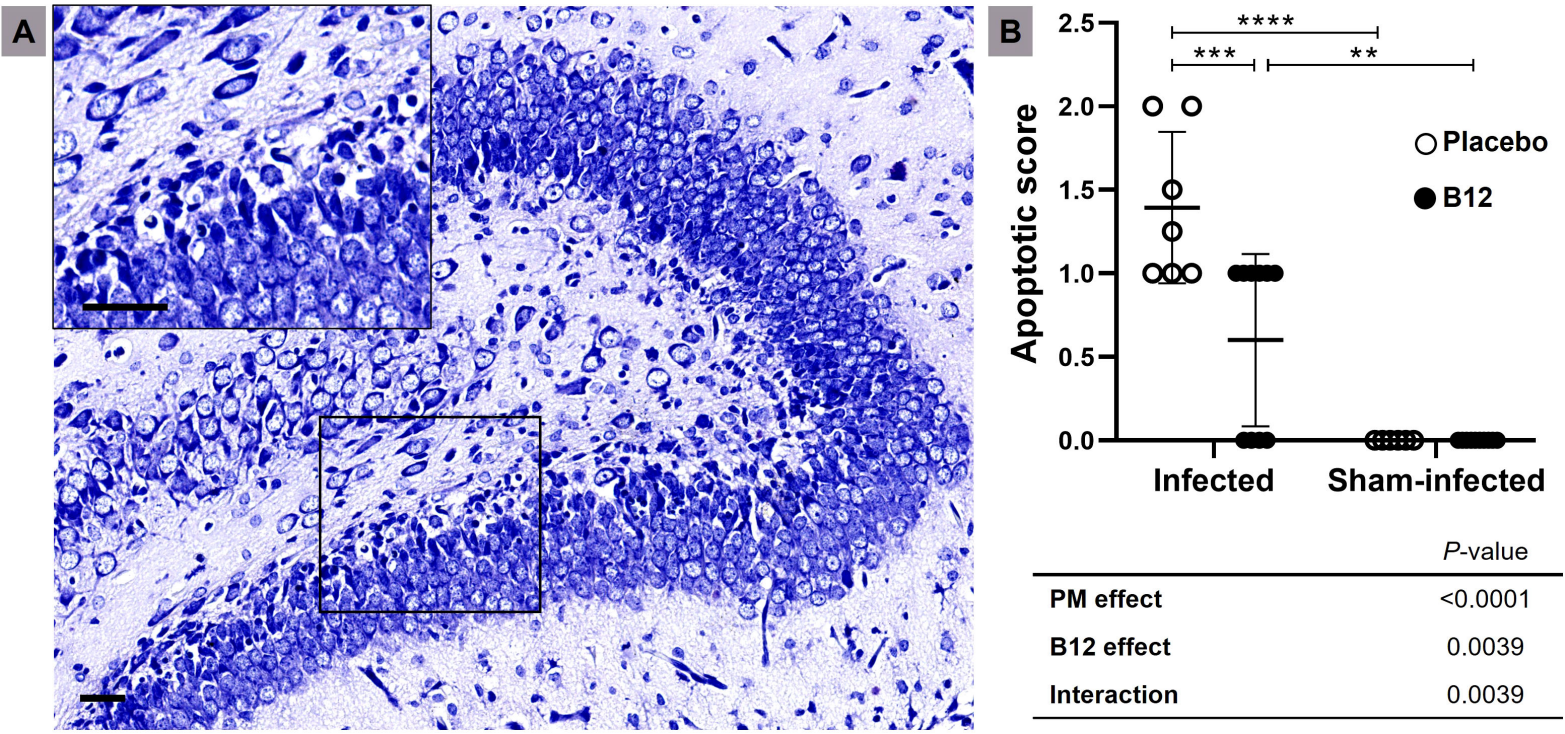
B12 treatment attenuates apoptosis in the granular layer of the dentate gyrus during PM. **(A)** Nissl-stained histological section showing the hippocampal dentate gyrus (10X) of an infected infant rat treated with placebo. The detail shows an amplified section of the lower blade of the granular layer (40X). Scale bar = 50 μm. **(B)** Apoptotic scores. Horizontal bars represent means with standard deviation. The effects of PM and adjuvant treatment with vitamin B12 were compared with two-way ANOVA followed by Tukey’s post-test. **P < 0.01; ***P < 0.001; ****P < 0.0001. PM, Pneumococcal meningitis.

Total RNA was extracted from hippocampi using Invitrogen Trizol reagent (Thermo Fisher) and chloroform (Merck), followed by purification with a miRNeasy Mini Kit column (Qiagen, Hilden, Germany) according to the manufacturer’s protocol. The concentration and integrity of the RNA samples were assessed using a Qubit 2.0 fluorometer (Thermo Fisher) and a Bioanalyzer 2100 (Agilent, Santa Clara, CA), respectively. Three animals from each group were chosen for RNA-Seq, based on their hippocampal apoptotic score values (**Figure 1**) being the closest to the medians of their respective groups. cDNA libraries were generated with the TruSeq Stranded mRNA Kit and sequenced using the TG Next-Seq 500/550 High Output Kit v2, 75 cycles (Illumina, San Diego, CA).

Raw reads were processed with Trimmomatic (6) to remove low-quality bases, and trimmed reads shorter than 50 nucleotides were discarded. Clean reads were then aligned to the *Rattus norvegicus* reference genome (release 94) using the software Spliced Transcripts Alignment to a Reference (STAR) (7). Read counts were quantified using only those located at a single genomic site. Contrast analyses between experimental groups (Sham-infected + B12 vs. Sham-infected + placebo; Infected + placebo vs. Sham-infected + placebo; Infected + B12 vs. Infected + placebo) were conducted using DESeq2 R package (8). Genes with a fold change greater than 1.5 and adjusted *P* values lower than 0.01 were considered differentially expressed (DEG) and were displayed in an UpSetPlot using the UpSetR R package (9). Functional enrichment analysis of the DEG was performed using Ingenuity Pathways Analysis (IPA) (Qiagen) (10) parameterized to use the Ingenuity Knowledge Base (genes + endogenous chemicals) considering direct and indirect relationships and the following filters: (species = Human OR Rat OR Mouse) AND (confidence = Experimentally Observed OR High (predicted)) AND (tissues/cell lines = Hippocampus OR Neurons OR all CNS Cell Lines OR CNS Cell Lines not otherwise specified OR Immune cells OR Hematopoietic progenitor cells). Deconvolution analysis of the RNA-Seq data to assess whether PM or adjuvant B12 affect the proportions of major leukocyte types in the inflammatory infiltrate was conducted using the CIBERSORTx software (11), specifically utilizing the “Impute Cell Fractions” module, and adjusting parameters for batch correction and number of permutations (100). Reference cell type-specific signatures were obtained from validated single-cell RNA-Seq data of rat-derived immune cells available in GitHub (RatDeconvolution) (12).

### 2.4 Validation of selected DEG by RT-qPCR

The High-Capacity cDNA Reverse Transcription Kit (Thermo Fisher) was used to synthesize cDNA following the manufacturer’s protocol. For qPCR reactions, Fast SYBR™ Green Master Mix (Thermo Fisher) was utilized with 1 ng/μL of cDNA in a final volume of 10 μL. Rat-specific primers were designed to detect putative biomarkers identified through transcriptome analysis, namely Neuronal PAS Domain Protein 4 (*Npas4*) (Forward 5’ CTCTCTTCCTGGCCATGTTC 3’; Reverse 5’ CTCCATTTTCAGCCAACAGG 3’) and Interferon Gamma (*Ifng*) (Forward 5’ CGCCAAGTTCGAGGTGAACA 3’; Reverse 5’ TTCCGCTTvCCTTAGGCTAGATTC 3’), using Primer-BLAST software (NCBI, Bethesda, MD, https://www.ncbi.nlm.nih.gov/tools/primer-blast/). Peptidylprolyl Isomerase A (*Ppia*) (Forward 5’ AGGATTCATGTGCCAGGGTG 3’; Reverse 5’ CTCAGTCTTGGCAGTGCAGA 3’) was employed as a reference gene for normalization of gene expression. The ViiA 7 real-time PCR system (Thermo Fisher) was used for thermal cycling and fluorescence detection, following the manufacturer’s recommendations. Relative expression of target genes was calculated using the 2^(−ΔΔCt) method (13).

### 2.5 Immunohistochemistry

The dewaxed slides (5 µm-thick coronal sections) were subjected to antigen retrieval through heat in Tris-EDTA buffer (pH 9.0) at 96°C for 40 minutes. Endogenous peroxidase activity was quenched with 3% hydrogen peroxide. To evaluate microglia activation and the intensity of the inflammatory infiltrate, a primary rabbit monoclonal antibody against the Ionized calcium-binding adaptor molecule 1 (Iba1) (Abcam, Cambridge, UK; #AB178847) was used at a dilution of 1:8000, along with the Novolink Polymer Detection System (Leica Biosystems, Nussloch, Germany) as per the manufacturer’s instructions. To assess histone epigenetic markings, non-specific protein binding was blocked with a commercial blocking reagent (EMD Millipore, Burlington, MA). Following blocking, the slides were incubated overnight at 4°C in a moist chamber with primary antibodies against trimethyl-Histone H3 (Lys4) (H3K4me3, rabbit polyclonal antibody, Merck #05-1339) and trimethyl-Histone H3 (Lys9) (H3K9me3, rabbit polyclonal antibody, Merck #07-442), diluted at 1:800 and 1:2000, respectively, in Rinse Buffer (EMD Millipore) plus 0.1% Tween 20 (Sigma-Aldrich). The visualization of antigen-antibody complexes was carried out using biotinylated goat anti-mouse IgG or anti-rabbit IgG, Streptavidin-HRP and 3’3 diaminobenzidine tetrachloride (EMD Millipore). After counterstaining with hematoxylin, the slides were dehydrated and mounted with a glass coverslip and xylene-based mounting media.

Images of histological sections were captured using the Aperio Scanner (Leica Biosystems) with a 40X objective and 3X, 20X, and 40X eyepiece. The captured images were subsequently processed using the Aperio ImageScope version 12.4.6 (Leica Biosystems) and Fiji (Image J) (14) software. The analysis of microglia activation in the lower blade of the dentate gyrus was performed utilizing the Skeleton Analysis method developed by Young et al. (15). Three images were analyzed for each animal. In this method, the data were represented by normalizing the endpoints (cell branch tips) in relation to branch lengths. To quantify the inflammatory infiltrate, the optical density of the leukocyte nuclei in the hippocampal fissure was measured. The resulting values were then normalized by fissure area.

For histone methylation analysis, images of the dentate gyrus were captured using the Leica DM5000B microscope (40X objective and 10X eyepiece), scanned using the Leica MC170HD camera, and processed with the Leica Application Suite program (Version 4.10.0). Immunohistochemically stained slides were subjected to morphometric analysis using the Leica Qwin V3, and the intensity of nuclear pixels (optical density) was identified in at least three images from each animal. For nuclear evaluation, the system was trained to recognize nuclei with a minimum pixel intensity corresponding to positive staining.

### 2.6 Statistical analysis

The statistical analyses were performed using GraphPad Prism version 6.0 (GraphPad Software Inc., Irvine, CA). Data distribution was assessed using Shapiro-Wilk test. Differences between groups were compared using the unpaired t test or two-way analysis of variance (ANOVA) with a Tukey’s or Bonferroni post-test to correct for multiple comparisons. Results were considered statistically significant if *P* values were less than 0.05.

## 3 Results

### 3.1 Clinical assessment and apoptosis in the lower blade of the dentate gyrus granular layer

Upon examination of the CSF at 18 hours post-infection, all animals infected with *S. pneumoniae* experienced PM with positive bacterial titers of approximately 1 × 10^8^ cfu/mL. Compared to sham-infected controls (mean ± standard deviation: 5 ± 0), infant rats with PM (3.917 ± 0.51) showed a significant decrease in activity score (*P* < 0.001), which was not affected by adjuvant therapy with vitamin B12 (3.818 ± 0.40). Histomorphological analysis confirmed that vitamin B12 had a neuroprotective effect (**Figure 1**).

### 3.2 Transcriptome analysis

#### 3.2.1 In healthy animals, vitamin B12 causes only minor changes in the hippocampal transcriptome

**Figure 2:**
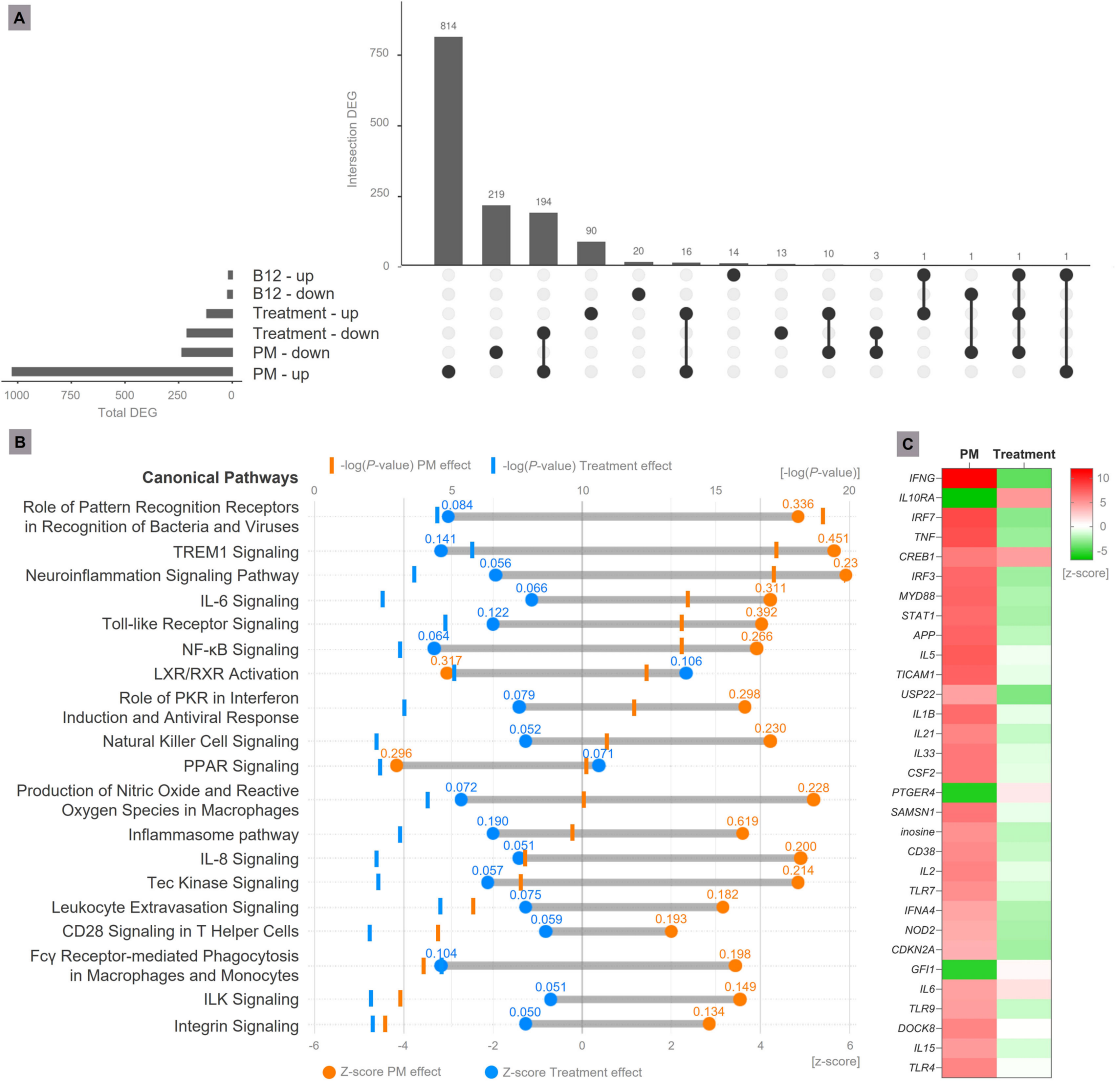
Transcriptome analysis. **(A)** Upset plot representing DEG in the analyzed contrasts (B12 effect = Sham-infected + B12 vs. Sham-infected + placebo; PM effect = Infected + placebo vs. Sham-infected + placebo; Treatment effect = Infected + B12 vs. Infected + placebo). Genes with Expression Log Ratio = -0.58 (down-regulated) or 0.58 (up-regulated) and Adjusted *P*-value < 0.01 were considered differentially expressed. **(B)** Main canonical pathways affected by PM and B12 treatment. During PM, the hippocampus transcriptome undergoes substantial changes, activating canonical pathways related to increased immune response and neuroinflammation (in orange). The treatment with vitamin B12 mitigates the major effects of PM (in blue), attenuating the pro-inflammatory profile. Values above the Z-scores represent the pathway ratio. **(C)** Main upstream regulator molecules modulated by PM and B12 identified in functional enrichment analysis. In red, predicted up-regulation; In green, predicted down-regulation. PM, Pneumococcal meningitis; DEG, Differently expressed genes; Suffixes: up, up-regulated; down, down-regulated.

The contrast between Sham-infected + B12 and Sham-infected + placebo disclosed 17 genes up-regulated and 21 genes down-regulated in healthy animals treated with the vitamin (**Figure 2A**). Although no canonical pathway was predicted to be affected, these 38 DEG are annotated with Gene Ontology (GO) terms predominantly related to positive regulation of glycolysis, gene expression, cell proliferation, neurotransmission, learning, memory, and inhibition of neuron differentiation and cell adhesion (**Additional File 1**).

#### 3.2.2 During PM, the hippocampus transcriptome undergoes substantial changes

In the hippocampus of animals with PM, 1025 genes were up-regulated and 234 were down-regulated (**Figure 2A**, **Additional File 2**). From this ensemble of DEG, several canonical pathways associated with increased immune response and neuroinflammation were predicted to be activated. Conversely, a few anti-inflammatory pathways, such as LXR/RXR Activation and PPAR Signaling, were predicted to be inhibited. The affected canonical pathways with the highest prediction confidence (*P* value) are shown in **Figure 2B**.

#### 3.2.3 B12 mitigates the major effects of PM on the hippocampus transcriptome

**Figure 3:**
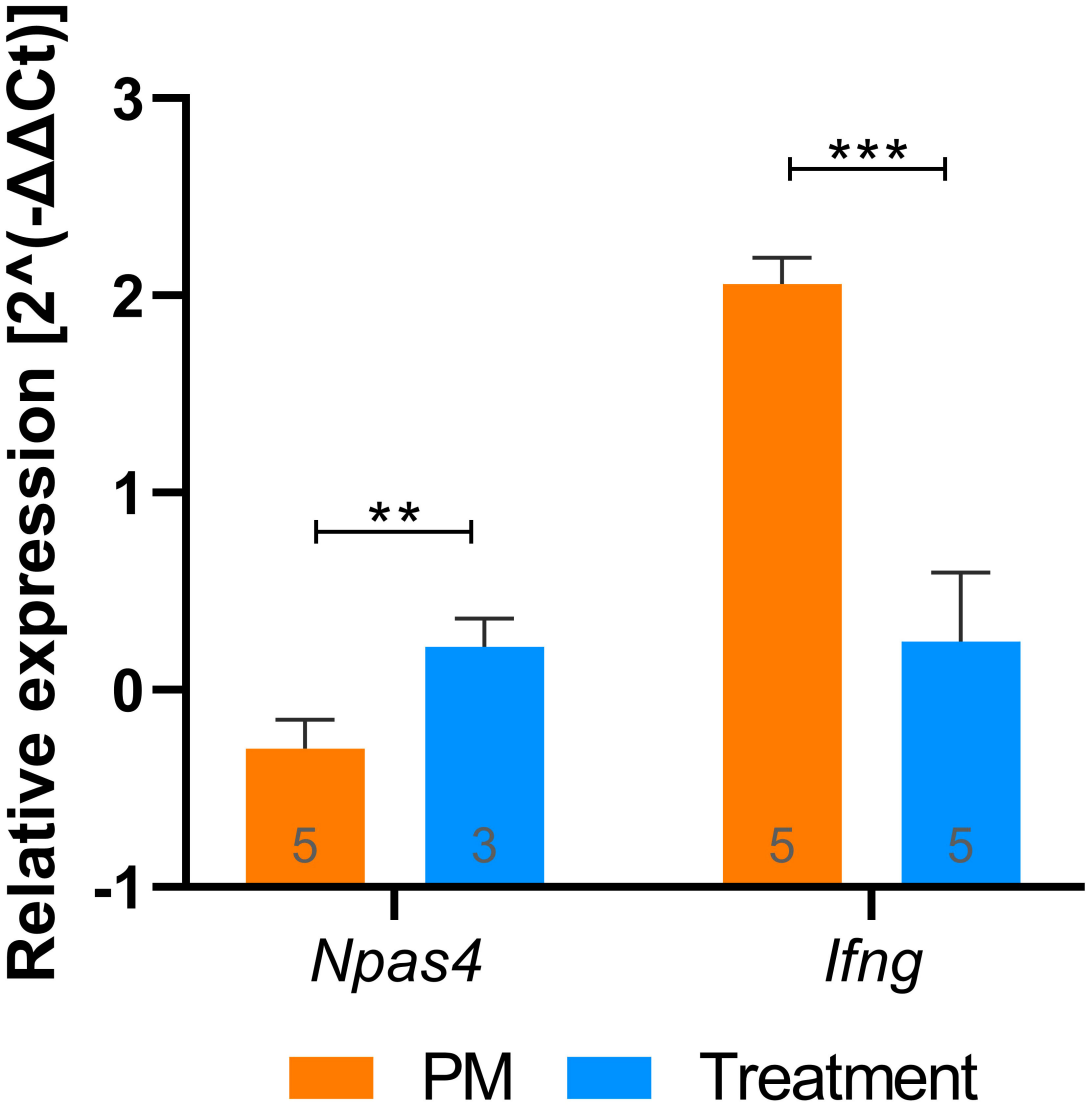
RT-qPCR validation of putative biomarkers identified with RNA-Seq. *Npas4* and *Ifng* relative expression values (2^(-ΔΔCt)) during PM and after treatment with B12. Groups were compared using unpaired t test. Outliers were identified by the ROUT test and removed from the analysis. Bars were expressed as mean with standard deviation. **P < 0.01; ***P < 0.001. PM, Pneumococcal meningitis.

In animals with PM treated with B12, 210 genes were down-regulated (194 were up-regulated by PM), and most of them are related to inflammation according to their GO annotations (**Figure 2A**, **Additional File 2**). This result is in line with the proven hypothesis that B12 inhibits gene expression by increasing the methylation of gene promoters (4). However, B12 up-regulated 118 genes, out of which 16 were also up-regulated by PM and 11 were down-regulated (**Figure 2A** and **Additional File 2**). An example of gene up-regulated by B12 in infected animals is *Npas4*. Its expression pattern was validated by RT-qPCR (**Figure 3**). These results indicate that B12 regulates gene expression in PM by another mechanism besides DNA methylation at gene promoters.

Functional enrichment analysis revealed that B12 counteracts the effect of PM in several canonical pathways related mainly to the immune response, oxidative stress, blood-brain barrier (BBB) integrity, and leukocyte migration (**Figure 2B** and **Additional File 3**). It is worth noting that the transcriptome analysis did not reveal any adverse effects of B12 in the context of PM. The main upstream regulator molecules modulated by PM and B12 were also identified with IPA (**Figure 2C**). The results clearly demonstrate that B12 counteracts the effects of PM in several upstream regulator genes with pivotal roles in inflammation and immune response and predicted with the highest confidence, as well as in functions related mainly to the infiltration of immune cells from the periphery to the CNS across the BBB. The effects of PM and B12 on the expression pattern of *Ifng*, the upstream regulator with the highest prediction confidence (**Figure 2C**), were validated by RT-qPCR (**Figure 3**).

#### 3.2.4 RNA-Seq deconvolution suggests that B12 attenuates the infiltration of neutrophils into the CNS

Based on RNA-Seq deconvolution analysis, PM is found to trigger the infiltration of neutrophils (PM effect: *P* = 0.0003 in two-way ANOVA) and, to a lesser extent, monocytes (PM effect: *P* = 0.0101 in two-way ANOVA) into the CNS. Additionally, this in silico approach suggests that B12 induces the infiltration of B cells into the CNS irrespective of the infection (PM effect: *P* = 0.0364 in two-way ANOVA). Notably, B12 significantly diminishes the infiltration of neutrophils into the CNS of infected animals (B12 effect: *P* = 0.0009 in two-way ANOVA; *P* < 0.001 when comparing Infect + placebo vs. Infect + B12 with Tukey’s post-test) (**Supplementary Material 4**).

### 3.3 Vitamin B12 attenuates hippocampal inflammation during PM

**Figure 4:**
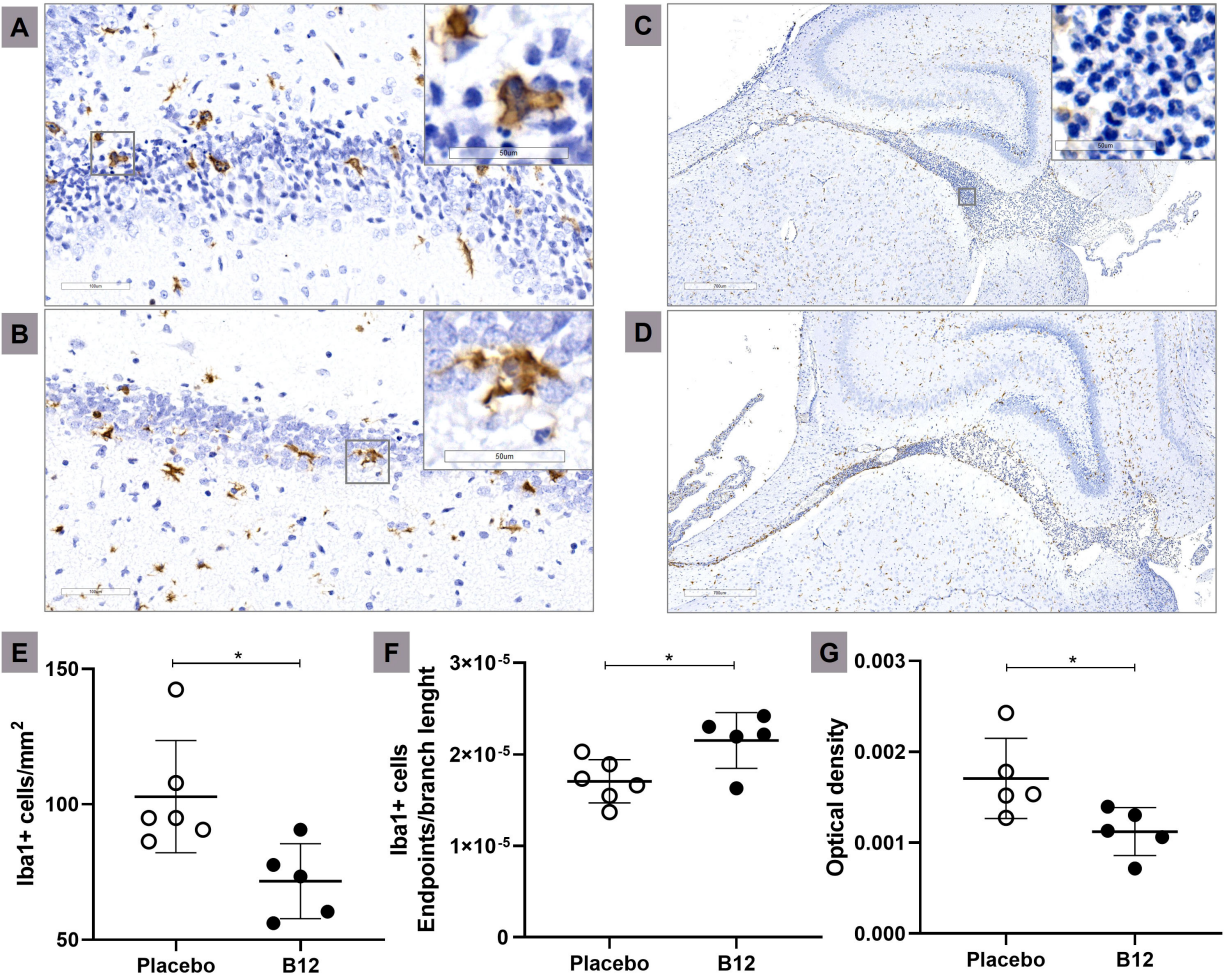
Microglia activation and inflammatory infiltrate during PM is attenuated in B12-treated infant rats. Immunohistochemical staining of Iba1^+^ cells (20X) of a representative infected animal administered with placebo **(A)** or B12 **(B)**. Scale bar = 100 μm. In detail, zooms (40X) of typical ameboid (more activated) and intermediate (less activated) microglia’s state, respectively Bar = 50 μm. Histological sections showing the hippocampal fissure (3X) of a representative infected animal administered with placebo **(C)** or B12 **(D)**. Scale bar = 700 μm. In detail, a zoom (40X) of the polymorphonuclear leukocytes present in the hippocampal fissure. Bar = 50 μm. **(E)** Microglia count (Iba1+ cells/mm^2^). **(F)** Microglia activation (Iba1^+^ cells endpoints\branch length). The highest values obtained corresponded to the less activated microglial state, indicating cells with a more branched morphology and smaller size. **(G)** Inflammatory infiltrate (Optical density). For all plots, horizontal bars represent means with standard deviation. The effects of PM and adjuvant treatment with vitamin B12 were compared with unpaired t test. *P < 0.05. PM, Pneumococcal meningitis.

Immunohistochemistry and histopathological analysis were conducted to evaluate microglia activation and the intensity of inflammatory infiltrate in the CNS during PM. **Figure 4** demonstrates that adjuvant B12 effectively alleviated microglia activation in the hippocampus of infected animals, as evidenced by the quantification of Iba1-expressing cells (Panel E) and the evaluation of cell morphology using the endpoints/branch length ratio (Panel F). Furthermore, B12 treatment reduced the inflammatory infiltrate in the hippocampal fissure (**Figure 4G**). These findings provide confirmation that adjuvant therapy with vitamin B12 attenuates PM-induced neuroinflammation, consistent with the predictions derived from the transcriptome analysis.

### 3.4 Histone methylation patterns

**Figure 5:**
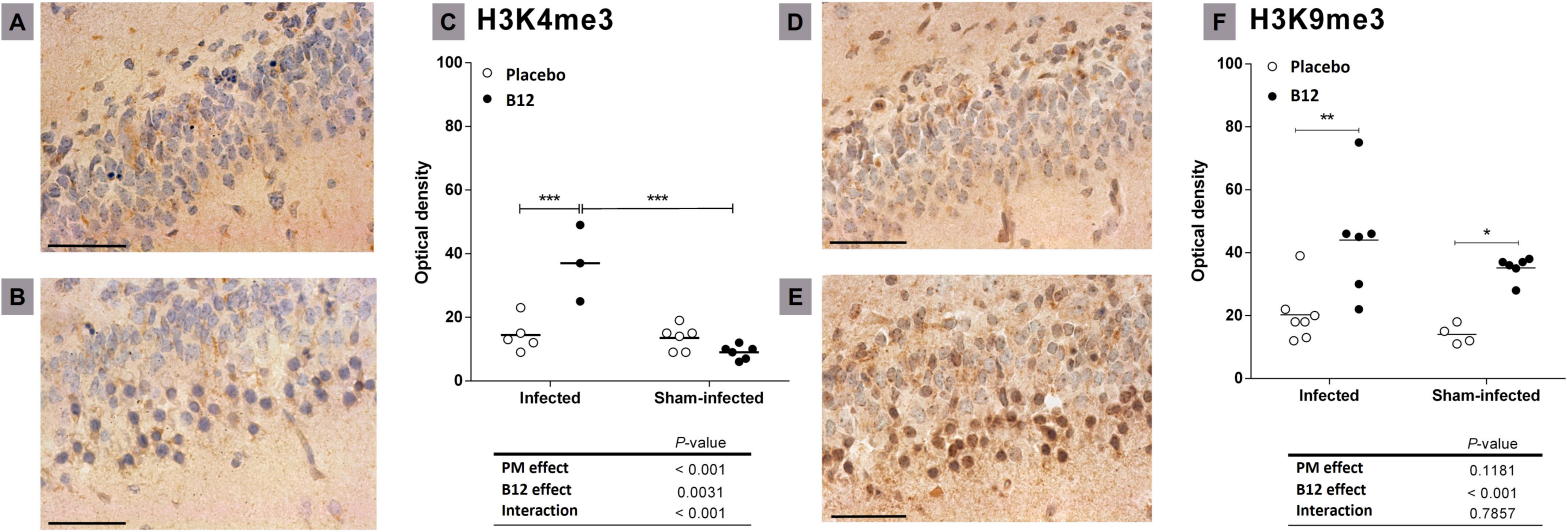
Effects of PM and B12 treatment on H3K4me3 and H3K9me3 in dentate gyrus granule cells. Histological sections showing the lower blade of the hippocampal granular layer (40X) immune-stained with antibodies anti-H3K4me3 **(A, B)**, or anti-H3K9me3 **(D, E)**. Scale bar = 100 μm. **(A, D)** infected rats administered with placebo. **(B, E)** infected rats treated with vitamin B12. **(C, F)** = Optical density. Horizontal bars represent means. The effects of PM and vitamin B12 were compared with 2-way ANOVA followed by Bonferroni post-test. *, *P* < 0.05; **, *P* < 0.01; ***, *P* < 0.001. PM, Pneumococcal meningitis.

Finally, immunohistochemically detectable changes in hippocampal patterns of histone H3 methylation induced by PM and/or vitamin B12 were assessed. PM did not affect the trimethylation of histone H3 at lysine 9 (H3K9me3) but significantly increased H3 trimethylation at lysine 4 (H3K4me3, *P* < 0.001) (**Figure 5**). In addition, adjuvant therapy with vitamin B12 in rats with PM increased the markings H3K9me3 (*P* < 0.001) and H3K4me3 (*P* < 0.01).

## 4 Discussion

The pathophysiology of PM involves a dynamic interplay between bacterial virulence factors and the host immune response, resulting in neuroinflammation and, ultimately, neurological dysfunction. The presence of the bacteria in the CSF initiates an inflammatory response, primarily mediated by microglia and perivascular and meningeal macrophages, which subsequently release inflammatory mediators such as cytokines and chemokines. These proinflammatory factors promote further inflammation and oxidative stress, leading to the disruption of the BBB, the pleocytosis of peripheral leukocytes, and the influx of excitatory amino acids into the CNS (2). This cascade of events triggers apoptosis in both progenitor cells and postmitotic neurons, which are scattered throughout the inner granular layer of the dentate gyrus in the hippocampus (16). This detrimental condition causes extensive damage to the hippocampal formation, a brain region which plays a crucial role in the consolidation of new memories and spatial navigation. Hippocampal apoptosis was associated with abnormal neuropsychological test results in animals after PM (17, 18). Besides, studies have shown that patients who have survive PM often experience long-term cognitive deficits, including problems with memory, attention, executive function, and cognitive performance (2, 19–21). Currently, PM is treated with antibiotics and corticosteroids, but these treatments do not prevent hippocampal damage (17, 18).

B12 vitamers are essential cofactors for key enzymes in energy metabolism and the sulfur amino acid pathway. They also play a beneficial role in neuroinflammatory conditions by scavenging reactive oxygen species (ROS), maintaining appropriate H_2_S levels, preventing the harmful effects of excess homocysteine, and reducing N-methyl-D-aspartate (NMDA) receptor-mediated excitotoxicity in CNS cells. Furthermore, B12 modulates chromatin remodeling mechanisms that down-regulate critical genes in neuroinflammation (22). It has been previously reported that adjuvant therapy with vitamin B12 mitigates apoptotic damage to the hippocampus and down-regulates some key inflammatory genes, namely *Il1b*, *Ccr2*, and *Ccl3*, in infant rats with PM (4). Specifically, vitamin B12 has been found to down-regulate the expression of *Ccl3* by enhancing the methylation of specific cytosine residues in its promoter region. Despite the fact that therapeutic administration of B12 has been shown to increase global DNA methylation in the hippocampus of healthy infant rats (4), the present study demonstrated that in the absence of infection, the vitamin regulates only a limited number of genes, which are expected to have an overall positive effect on hippocampal function (**Additional File 1**). Therefore, the findings of the present study provide support for the expected safe profile of B12.

PM is known to broadly modify the hippocampal transcriptome, leading to the activation of several pathways functionally related to the major components of the physiopathology of this disease, including microglia activation, oxidative stress, BBB disruption, leukocyte extravasation into the CNS, neuroinflammation, and cell death, among others. Furthermore, PM also inhibits some anti-inflammatory and cell survival pathways (23, 24). The findings of the present study support and improve this previous knowledge. Of particular interest in the scope of this study is the outcome of adjuvant therapy with B12 in the canonical pathways most affected by PM, as the vitamin effectively reversed these disease effects (**Figure 2B**, **Additional File 3**).

Pivotal canonical pathways involved in pneumococci detection in the CNS and signaling to trigger the innate immune response, namely *Role of PRRs in Recognition of Bacteria and Viruses*, *Toll-like Receptor signaling*, *Inflammasome pathway*, *NF-kB Signaling*, and *TREM1 Signaling*, are activated by PM, and inhibited by B12 (**Figure 2B**, **Additional File 3**).

The first line of defense against invading pneumococci in the CNS is constituted by the innate immune system, which is activated by the recognition of conserved microbial structures or pathogen-associated molecular patterns (PAMPs) by antigen presenting cells (APC) expressing pattern-recognition receptors (PRRs) (25). Among these APCs there are blood cells, which are predominantly found in the meninges, choroid plexus, and perivascular space, as well as astrocytes and microglial cells that reside within the brain parenchyma (26). Transmembrane Toll-like receptors (TLRs) 2, 4, and 9, and cytosolic Nucleotide-Binding Oligomerization Domain 2 (NOD2) receptors are critical PRRs responsible for APC detection of *S. pneumoniae* in the CNS (27–29). All TLR as well as NOD2 signaling pathways ultimately result in the activation of Nuclear Factor Kappa B (NF-kB) (28, 30). This transcriptional factor activates several genes that contribute to the pathophysiology of BM, such as IL-1β, Tumor Necrosis Factor (TNF)-α, Interleukin 6 (IL-6), Interleukin 8 (IL-8), Macrophage Inflammatory Protein-1 Alpha (MIP-1α), inducible nitric oxide synthase (iNOS), cyclooxygenase-2 (COX)-2, and Intercellular Adhesion Molecule-1 (ICAM-1) (31). Additionally, the Triggering Receptor Expressed On Myeloid Cells (TREM) protein receptor family is emerging as a crucial regulator of various cellular functions, including inflammation amplification. Evidence indicates that activation of TREM-1 through danger- and pathogen-associated molecular patterns (DAMPs and PAMPs) can lead to the production of cytokines (32). TREM-1 signaling has already been associated with host defense during the early stages of infection with highly pathogenic *Streptococcus suis* (33). Inflammasome-mediated recognition of the pneumococcus also contributes to the host innate immune response. Indeed, the NALP3 inflammasome regulates Caspase-1 activation and subsequent secretion of both IL-1β and Interleukin 18 (IL-18) (34). NK cells stimulated by IL-18 produce the pro-inflammatory cytokine Interferon Gamma (IFNG), which is a pivotal driver of neuropathology and behavioral sequelae in experimental PM. IFNG modulates a range of processes, including myeloid recruitment and activation, as well as inhibition of bacterial clearance (35). Although the levels of IFNG are elevated in both the CSF of patients with PM (36, 37) and in the brain tissue of rats (38) with the disease, the exact role of this cytokine in PM remains unclear. The present study identified IFNG as the upstream regulator of several critical genes regulated by PM. It is noteworthy that B12 significantly mitigated the impact of the infection on these IFNG-regulated genes. Furthermore, B12 counteracted the PM-induced alterations in the expression of multiple genes regulated by other predicted upstream regulatory molecules (**Figure 2C**).

In addition to counteracting the effects of PM in the initial processes of pathogen recognition and innate immunity activation, B12 also inhibits disease effects in canonical pathways that directly regulate the major component of neuroinflammation (*Neuroinflammation Signaling Pathway, IL6 Signaling*, *Integrin Signaling*, and *Leukocyte Extravasation Signaling, Production of NO and ROS in macrophages*, and *Fcy Receptor-mediated Phagocytosis in Macrophages and Monocytes*) (**Figure 2B**, **Additional File 3**). Neuroinflammation is defined as an inflammatory response within the brain or spinal cord (39). This response is mediated by the production of cytokines, chemokines, ROS, reactive nitrogen species (RNS), and secondary messengers produced by microglia, astrocytes, endothelial cells, and peripherally derived immune cells.

The activation of NF-kB through PRRs stimulates the production of early-phase cytokines, IL-1β, TNF, and IL-6 (40, 41). These cytokines induce the up-regulation of several adhesion factors on the vascular endothelium, leading to the influx of leukocytes, primarily neutrophils, into the infected site, mainly within the first few hours of infection (42–45). However, in PM, it appears that IL-6 is not directly associated with promoting pleocytosis in the CSF but rather with brain edema, BBB disruption, and increased intracranial pressure (46). Leukocytes cross the BBB by binding to selectins and rolling across the endothelium (45). Integrins up-regulated on the vascular endothelium facilitate the binding of leukocytes and subsequent BBB migration (26).

During PM, endothelial cells and neutrophils produce RNS such as nitric oxide (NO), catalyzed by endothelial nitric oxide synthase (eNOS) and iNOS, respectively (47, 48). Neuronal nitric oxide synthase (nNOS) appears to play a minor role in BM (49). Moreover, in neutrophils, macrophages, and endothelial cells, NADPH oxidase induces the production of ROS, such as superoxide (O_2_^-^), in response to infection (50). Although neutrophils produce a higher amount of ROS compared to macrophages, the latter cells produce a greater quantity of RNS than the former (51). *S*. *pneumoniae* generates hydrogen peroxide, which reacts with NO to produce peroxynitrite (ONOO^-^). This highly reactive compound can cause lipid peroxidation and destabilization of cell membranes, DNA damage, and subsequent activation of the DNA repair enzyme poly (ADP-ribose) polymerase (PARP) leading to cellular energy collapse and death (48, 50). Moreover, ROS and RNS have been identified as mediators of BBB breakdown (52).

Phagocytosis is a host cell endocytic response to particulate matter like bacteria. The process of phagocytosis is complex and comprises several events like particle binding, receptor clustering, actin nucleation, pseudopod extension, membrane recycling, and phagosome closure. The Fc gamma receptors (FcγR; subtypes FcγR1A, FcγRIIA and FcγRIIIA) of the immunoglobulin superfamily are the best-characterized receptors for phagocytosis in avidly phagocytic cells of the hematopoietic lineage, like macrophages, neutrophils, and microglia (53, 54).

The findings discussed above are in line with the results of a previous study in which B12 was shown to down-regulate proinflammatory genes that are up-regulated by PM by promoting the hypermethylation of their promoter regions (4). However, B12 also activated the anti-inflammatory canonical pathways *PPAR Signaling* and *LXR/RXR Activation*, which are inhibited by PM.

The peroxisome proliferator-activated receptors (PPARs) are a group of nuclear receptor proteins that regulate the transcription of genes involved in energy production, lipid metabolism, and inflammation. The PPAR family includes PPARα and PPARγ. Upon activation by their ligands, these two receptors down-regulate the production of pro inflammatory cytokines such as TNF, IL-6, and Il-1β. In the case of PPARα, these effects are due to its capacity to inhibit NF-κB signaling pathway (55).

The Liver X receptors (LXRs) are a group of nuclear receptor proteins that play a significant role in the regulation of lipid metabolism and cholesterol homeostasis, including the conversion of cholesterol to bile acids. LXRs have also been shown to modulate immune and inflammatory responses, particularly in macrophages. LXRs act as ligand-dependent transcription factors that form heterodimers with the retinoid X receptor (RXR), which then bind to LXR-responsive elements (LXREs) in DNA to promote the expression of target genes. Interestingly, ligand activation of LXRs not only activates transcription of target genes, but also inhibits transcription from promoters of certain genes of proinflammatory cytokines that do not contain LXREs, a phenomenon referred to as trans-repression (56). Due to their cholesterol sensing and anti-inflammatory activities, LXRs are considered as integrators of metabolic and inflammatory signaling (57).

The anticipated anti-neuroinflammatory and neuroprotective effects of B12, as predicted through transcriptome analysis in infected animals treated with the vitamin, were successfully confirmed at the histological level (**Figures 4** and **1**, respectively). The anti-inflammatory effect of B12 is further supported by the deconvolution analysis of the RNA-Seq data, suggesting that B12 reduces the infiltration of neutrophils into the CNS in infected animals. These computational findings also align with prior research, which demonstrated that PM triggers the infiltration of neutrophils, and to a lesser extent, monocytes, into the CNS (58). The suggested increase in the B cell fraction within the inflammatory infiltrate in the CNS due to B12, regardless of the infection, warrants further investigation. It is important to note that these results should be interpreted cautiously since the reference dataset does not include microglial cells.

In infected animals, adjuvant therapy with B12 leads to the up-regulation of another interesting gene, *Npas4* (**Supplementary Material 2**, **Figure 3**). *Npas4* is a transcription factor with pronounced expression in the brain, including the hippocampus, where it plays a pivotal role in regulating the formation and maintenance of inhibitory synapses in response to excitatory synaptic activity (59, 60). By this mechanism, *Npas4* may act as a neuroprotective factor in the context of PM, where the influx of excitatory amino acids into the CNS can induce neuronal excitotoxicity (61). A previous work demonstrated that administering B12 to infant rats with PM increases methyl bioavailability and DNA methylation in the hippocampus (4). However, the observed expression pattern of *Npas4*, as further validated by RT-qPCR (**Figure 3**), cannot be attributed to an increase in its promoter methylation induced by B12. To explore potential explanations for the positive regulation of genes like *Npas4* by B12, histone markings were assessed by immunohistochemistry in hippocampal sections. The up-regulation of *Npas4* in PM animals treated with B12 indeed correlates with the expected effect of the increased epigenetic mark H3k4me4 observed within the granular layer of the hippocampal dentate gyrus of animals in this particular group (**Figure 5**). H3k4me3 is associated with the active transcriptional state of chromatin, while H3k9me3 is linked to transcriptionally inactive heterochromatin (62). Thus, the results presented herein suggest that changes in histone methylation patterns may work in concert with DNA methylation, ultimately contributing to a positive balance in protecting progenitor cells and postmitotic neurons in the hippocampal dentate gyrus during PM, as depicted in **Figure 1**.

It is conceivable that the favorable impact of therapeutic vitamin B12 in PM may also be partially mediated by its direct effects in counteracting oxidative stress, preventing excitotoxicity, and other related mechanisms. This study was limited by its sole focus on exploring the impact of the vitamin on epigenetic and transcriptional regulation.

## 5 Conclusion

In conclusion, adjuvant therapy with B12 modulates the hippocampal transcriptional signature induced by PM in a way that is consistent with the mitigation of several aspects of the innate immune response activated by the disease. These aspects include the recognition of pathogens by immune system cells, signaling via NF-kB, production of pro-inflammatory cytokines, migration of peripheral leukocytes into the CNS, and production of ROS and RNS. B12 also activates anti-inflammatory pathways inhibited by PM. Consequently, B12 attenuates neuroinflammation and apoptotic cells death in the hippocampal dentate gyrus. The effects of B12 at the transcriptional level are mediated not only through the previously demonstrated DNA hypermethylation but also by alterations in histone methylation patterns. Notably, no adverse effects of B12 were predicted or observed, reinforcing the well-known safety profile of this epidrug. These findings support the proposition of a clinical trial to evaluate the potential of B12 as an adjuvant therapy to mitigate hippocampal damage associated with neuroinflammation in PM.

## Data availability statement

The datasets presented in this study can be found in online repositories. The names of the repository/repositories and accession number(s) can be found below: https://www.ncbi.nlm.nih.gov/sra/PRJNA966183.

## Ethics statement

The animal study was approved by Ethics Committee on the Care and Use of Laboratory Animals (CEUA-FIOCRUZ, protocol LW-23/17). The study was conducted in accordance with the local legislation and institutional requirements.

## Author contributions

LC - performed most of the experiments, analyzed and interpreted data, and wrote the manuscript. MO – contributed to the experiments, analyzed and interpreted data, and critically reviewed the manuscript. KQ - contributed to the experimental infection and immunohistochemistry, and reviewed the manuscript. AA - contributed to the immunohistochemistry, and reviewed the manuscript. AS - made substantial contributions to the NGS experiments; GF - made substantial contributions to the Bioinformatic analysis, and reviewed the manuscript. CC - made substantial contributions to the histological and immunohistochemical analysis, analyzed and interpreted data, and critically reviewed the manuscript. RC - designed and oversaw the study, analyzed and interpreted data, and wrote the manuscript. All authors contributed to the article and approved the submitted version.
