# Supplementary material for: Uncovering the neuroprotective effect of vitamin B12 in pneumococcal meningitis: insights into its pleiotropic mode of action at the transcriptional level
Source: Front Immunol. 2023 Oct 3;14:1250055. doi: 10.3389/fimmu.2023.1250055 (PMC10579599; doi:10.3389/fimmu.2023.1250055)
Supplement: Supplementary file 4 [file DataSheet_4.docx]

**Supplementary Material 4**: Deconvolution analysis





(**A**) Relative cell fractions normalized to 1 across analyzed immune cell-types. (**B**) to (**G**) Cell fractions in each experimental group. For all plots, horizontal bars represent means with standard deviation. The effects of PM and adjuvant treatment with vitamin B12 in cell fractions were compared with two-way ANOVA followed by Tukey’s post-test. ****P* < 0.001. Abbreviations: PM = Pneumococcal meningitis.
